# A flavonoid rich standardized extract of Glycyrrhiza glabra protects intestinal epithelial barrier function and regulates the tight-junction proteins expression

**DOI:** 10.1186/s12906-021-03500-1

**Published:** 2022-02-07

**Authors:** Sasi Kumar Murugan, Bharathi Bethapudi, Subramanian Raghunandhakumar, Divya Purusothaman, Muruganantham Nithyanantham, Deepak Mundkinajeddu, Muralidhar Srinivasaih Talkad

**Affiliations:** 1 Department of Pharmacology & Toxicology, R&D Centre, Natural Remedies Private Limited, Plot No. 5B Veerasandra Indl. Area 19th K. M. Stone Hosur road Electronic City Post, 560100 Bengaluru, Karnataka India; 2 Department of Pharmacology, Saveetha Dental College & Hospitals, Saveetha Institute of Medical and Technical Sciences (SIMATS), Chennai, Tamil Nadu India; 3 Department of Animal Health Science, R&D Centre, Natural Remedies Private Limited, Bengaluru, Karnataka India

**Keywords:** Leaky gut, *Glycyrrhiza glabra*, Intestinal permeability, 2,4,6-Trinitrobenzenesulfonic acid, TNF-alpha, Tight junction proteins

## Abstract

**Background:**

Intestinal epithelial barrier dysfunction predisposes to many gastrointestinal, metabolic, and psychological disorders. A flavonoid rich extract of *Glycyrrhiza glabra* (FREG) has previously been reported to possess anti-inflammatory, antioxidant, and antiulcer properties.

**Aim:**

To investigate the effect of FREG (GutGard^®^) on restoring intestinal barrier function in tumor necrosis factor-alpha (TNF-α) stimulated human colonic adenocarcinoma cell monolayer (Caco-2) and 2,4,6-Trinitrobenzenesulfonic acid (TNBS) induced ulcerative colitis in rats.

**Methods:**

In in vitro, human intestinal Caco-2 cell monolayers were treated with TNF-α in the presence or absence of FREG and the paracellular permeability to FITC-conjugated 4-kD dextran (FD4) was measured to evaluate protection against the barrier dysfunction. In in vivo, intestinal barrier dysfunction was induced in male albino Wistar rats via intrarectal instillation of TNBS. Subsequently, the rats were treated orally with either FREG at 6.25, 12.5, and 25 mg/kg body weight, or Mesacol (250 mg/kg) for 5 days. On day 5, intestinal epithelial permeability was assessed with FD4 leakage into the serum. Also, colonic inflammation, colon morphology, histology and macroscopic score, weight to length ratio were evaluated. The activity of myeloperoxidase (MPO), TNF- α, secretory IgA levels and tight junction proteins expression were evaluated in rat’s colon.

**Results:**

FREG protected the intestinal epithelial barrier integrity in human intestinal Caco-2 cells in vitro. FREG administration significantly improved the intestinal epithelial barrier function as evident from significant reduction in FD4 leakage. The colon morphology, histology score, macroscopic score, colon weight to length ratio also indicates beneficial effects of FREG on barrier function. In addition, FREG regulated the tight junction proteins, and markedly decreased TNF-α, MPO levels and significantly increased the secretory IgA levels in TNBS induced colitis rats.

**Conclusion:**

The study findings support the protective action of FREG on intestinal epithelial barrier integrity indicating its potential in protecting from implications of leaky gut.

**Supplementary Information:**

The online version contains supplementary material available at 10.1186/s12906-021-03500-1.

## Introduction

The intestinal epithelium is constituted of a single layer of columnar epithelial cells (IECs) that forms a physical and functional barrier to prevent the entry of toxins, commensal and pathogenic microorganisms into the underlying mucosa and contacting with the immune cells. IECs are tightly bound together by tight junctions and its associated proteins, such as occludin, zonula occludens (ZO) and claudins which are critical to the maintenance of gut barrier integrity [1, 2]. If any abnormalities occur in the tight junctions and its associated proteins, the epithelial integrity may get compromised, which is termed as leaky gut. A leaky epithelium allows the entry of luminal antigens from the gut lumen into the systemic circulation, which may further promote both local and systemic immune responses [3]. The gut barrier integrity is frequently disrupted in a variety of acute or chronic intestinal diseases, such as ulcerative colitis, Crohn's disease, irritable bowel syndrome (IBS), celiac disease and infectious diarrhoea [4–8]. These diseases induce the release of proinflammatory cytokines (TNF-α, INF-γ, IL-1β, IL-6, IL-13), increased the oxidative stress by release of ROS and contribute to the intestinal barrier dysfunction or leaky gut [9–14]. A variety of animal models have been developed to evaluate the efficacy of therapeutic and prophylactic agents on intestinal barrier dysfunction [15]. Intrarectal instillation of 2,4,6- trinitrobenzene sulfonic acid (TNBS) is a well-established and widely used model for inducing intestinal barrier dysfunction in rats.

Plants have been used as the major source of medicine from ancient times. In the traditional system of medicine, various indigenous plants are being used for prevention, and elimination of various gastrointestinal disorders. There is an increasing demand for plant-based medicines, health products, pharmaceuticals, and food supplements across the world due to their safety, efficacy, and lesser side effect [16]. *Glycyrrhiza glabra* Linn. (Family: Fabaceae), commonly known as licorice, is a well-known medicinal plant in traditional system of medicine globally for its ethnopharmacological value to cure various ailments. The roots and stolons are the main medicinal parts of licorice and shown to possess various medicinal properties like antitussive, antimicrobial, hepatoprotective, antiadhesive, anxiolytic, expectorant, antioxidant, anti-inflammatory, antiulcer, anticancer & etc. due to the presence of bioactive components such as triterpene, saponins, flavonoids, alkaloids, glycyrrhizin, glycyrrhetic acid, glabridin, liquiritin etc. [17]. Among the bioactive compounds of *G. glabra*, flavonoids contribute significantly to the biological activities reported.

FREG is a standardized deglycyrrhizinated flavonoid rich root extract of *Glycyrrhiza glabra*, the safety and efficacy of which was evaluated in our earlier studies. FREG did not show any evidence of clastogenic and mutagenic potential in a battery of in vitro genotoxicity tests [18] and it was found to be safe up to 5000 mg/kg rat body weight in the acute oral toxicity study. In two randomized double-blind placebo-controlled clinical studies on functional dyspepsia & *H.pylori*, FREG administration to 60 days was found to be effective, safe and tolarable [19, 20]. Furthermore, FREG exhibited anti-inflammatory activity possibly through the inhibition of COX and LOX pathways in in vitro systems [21]. A dose dependent antioxidant and antiulcer activity was demonstrated in various ulcer models [22]. In addition, LPS induced proinflammatory/inflammatory mediators were significantly inhibited by FREG in murine macrophages [23]. FREG has significant anti-inflammatory, antioxidant and anti-ulcer benefits. With this background, we investigated the actions of FREG on protecting the barrier function in TNBS induced colitis rats and TNF-alpha induced human intestinal Caco-2 cells.

## Materials and Methods

### Test substance

The investigational test substance GutGard^®^ is a flavonoid rich, standardized root extract of *Glycyrrhiza glabra* developed by Natural Remedies Pvt Ltd, Bangalore, India. Gutgard is standardized to ≥ 10% w/w total flavonoids by HPLC including ≥ 3.5% w/w of glabridin.

### In vitro intestinal barrier dysfunction study

#### Chemicals, Reagents & Instruments

Dulbecco's Modified Eagle Medium (DMEM) and Fetal bovine serum (FBS) were purchased from GIBCO Laboratories, UK; 3-(4,5-dimethylthiazol-2-yl)-2,5-diphenyltetrazolium bromide (MTT) and Phosphate buffered saline (PBS) were purchased from Sigma-Aldrich, USA; Fluorescein isothiocyanate-dextran (FITC-d/FD4) was purchased from Sigma Aldrich, Sweden; TNF-alpha was procured from BioVision, USA. The instruments used were CO_2_ incubator from Kendro, Germany and Microplate reader from Molecular devices, USA.

### Cell culture conditions

Human Caucasian colorectal adenocarcinoma (Caco-2) cell lines were obtained from American Type Culture Collection (ATCC) (Rockville, MD, USA). Caco-2 cells was cultured in DMEM containing 10% (v/v) FBS and 1% (v/v) penicillin–streptomycin antibiotics. Cells were maintained in a humidified incubator at 37 °C in an atmosphere of 5% CO_2_ and were subcultured once a week with fresh culture medium. The confluent Caco-2 cells were detached with trypsin, counted, and seeded at a density of 1 × 10^4^ cells per mL onto a Transwell cell culture inserts system with 0.4 μm pore size (Merck Millipore, USA). Cells were then cultured for 19–21 days to reach confluent monolayer with the growth media which was replaced every alternative day. The transepithelial electrical resistance (TEER) values were measured using millicell and Caco-2 cell monolayers with the indicative TEER value of greater than 450 Ώ cm^2^ were used for further experiments [24].

### Cytotoxicity assay

Cytotoxicity of FREG in Caco-2 cells was measured by 3-(4,5-dimethylthiazol-2-yl)-2,5-diphenyltetrazolium bromide (MTT) assay. Caco-2 cells were harvested and transferred into 96-well microplates (1 × 10^4^ cells/well). After incubation, cells were exposed with increasing concentrations (2.5–40 μg/mL) of FREG for 48 h at 37 °C in a humidified incubator (5% CO_2_, 95% air). Thereafter, the cells were washed and incubated for 1 h with 10 µl of MTT (5 mg/mL). Consequently, the supernatant was removed, and 200μL of dimethyl sulfoxide was used to dissolve the formazan crystal. The cell viability was calculated by reading the absorbance of each well at 570 nm using a microplate reader [24].

### Effect of FREG in protecting the intestinal barrier function

Caco-2 cells were washed with pre-warmed PBS and then FREG (10, 12.5 and 15 µg/mL) were added to the apical compartment. Consequently, Caco-2 cells were treated with TNF-α (20 ng/mL) from the basolateral compartment to induce intestinal barrier dysfunction and incubated for 24 h in a humidified incubator at 37 °C with 5% CO_2_. The protective effect of FREG on the intestinal barrier dysfunction was estimated by the amount of FD4 leakage [25].

### Effect of FREG in restoring the intestinal barrier function

Caco-2 cells were washed with pre-warmed PBS and then antibiotic free DMEM containing TNF-α was added to the basolateral compartment. After 24 h incubation, the Caco-2 cell monolayers were washed on both the apical and basolateral sides with pre-warmed PBS. Subsequently, the apical compartment of the Caco-2 cells was incubated with FREG (10, 12.5 and 15 µg/mL) for 24 h in 5% CO_2_ incubator at 37 °C. The FD4 leakage was measured to assess the restoration of intestinal barrier function [25].

### In vivo TNBS- induced intestinal barrier dysfunction study

#### Chemicals, Kits & Instruments

Picrylsulfonic acid/TNBS was purchased from Sigma Aldrich, USA; FITC-d/FD4 was purchased from Sigma Aldrich, Sweden; Carboxymethyl cellulose (CMC) and Sodium chloride (NaCl) were purchased from Himedia Laboratories Pvt Ltd, India; Ethanol from Merck, Germany; Mesacol drug from Sun Pharma Laboratories Ltd, India were obtained.

Myeloperoxidase and secretory IgA assay kits were purchased from BT Bioassay Technology Laboratory, China; TNF-alpha assay kit was purchased from Cusabio, USA; Lysis buffer from Cell Signaling Technology Inc, USA; BCA protein assay kit from Thermo Scientific, USA; Polyvinylidene difluoride (PVDF) membranes from Hybond- Amersham, USA; The primary antibodies of tight junction proteins such as claudin-2, occludin, zonula occludens-1 (ZO-1 or TJ protein-1) were purchased from Invitrogen, USA and secondary antibody from Santa Cruz Biotechnology, USA; Chemiluminescence detection kit from Bio-Rad, USA. The instruments used were Microplate reader from Molecular devices, USA; ChemiDoc image scanner from Bio-Rad, USA.

## Animals

Male albino Wistar rats weighing 220–240 g were obtained from R&D Centre, Natural Remedies Pvt Ltd, Bangalore, India. The rats were acclimatized for 7 days prior to the experimentation and maintained under standard housing conditions such as 12 h light/ 12 h dark cycle, temperature at 23 ± 2 °C, with a relative humidity of 30—70%. Animals had free access to standard pellet feed (M/s Amrut Laboratory Animal Feeds, India) and UV purified water ad libitum throughout the experimental period. All the experimental protocols were approved by Institutional Animal Ethics Committee (IAEC) of Natural Remedies Pvt Ltd, Bangalore, India (Approval no.: IAEC/NR-PCL-02/08/19) and experiments were conducted according to the Committee for the Purpose of Control and Supervision of Experiments on Animals (CPCSEA) and in compliance with the Animal Research: Reporting of In Vivo Experiments (ARRIVE) guidelines.

### Experimental Procedure

#### (1) Induction of intestinal barrier dysfunction by TNBS and drug treatment schedule

Intestinal barrier dysfunction was induced according to the procedure described by Liu et al., 2016 & Guo et al., 2020 [26, 27]. Overnight fasted animals were anesthetized with isoflurane, and then TNBS, dissolved in 50% ethanol, was instilled into the colon of the animals (100 mg/kg in a volume of 0.75 mL) using a medical-grade polyethylene catheter (PE-90). It is inserted about 8 cm into the anus. After instillation, the animals were supported in a supine position until recovery from anaesthesia to prevent the immediate anal leakage of TNBS. TNBS was administered as a single dose, FREG or Mesacol were given orally for 5 days. Animals were randomly allocated into the following groups with 6 animals in each group:

1. ***Group I***: Normal control animals: Received 0.75 mL of physiological saline (Once; intrarectally) and were administered with 0.5% CMC (10 ml/kg; *p.o*.) for 5 days.
2. ***Group II***: TNBS control animals: Received 0.75 mL of TNBS (Once; 100 mg/kg; intrarectally) and were administered with 0.5% CMC (10 ml/kg; *p.o.*) for 5 days.
3. ***Group III***: Mesacol (Mesalamine) treated animals: Received 0.75 mL of TNBS (Once; 100 mg/kg; intrarectally) and were treated with Mesacol (250 mg/kg; *p.o.*) for 5 days.
4. **Group IV**: FREG treated animals: Received 0.75 mL of TNBS (Once; 100 mg/kg; intrarectally) and were treated with FREG (6.25 mg/kg; *p.o.*) for 5 days.
5. ***Group V***: FREG treated animals: Received 0.75 mL of TNBS (Once; 100 mg/kg; intrarectally) and were treated with FREG (12.5 mg/kg; *p.o.*) for 5 days.
6. ***Group VI***: FREG treated animals: Received 0.75 mL of TNBS (Once; 100 mg/kg; intrarectally) and were treated with FREG (25 mg/kg; *p.o.*) for 5 days.

#### (2) Measurement of intestinal barrier permeability

On day 5, after the last dose of treatment, rats were denied access to food but were allowed water for 4 h for the measurement of intestinal barrier function/permeability using a permeability marker (FD4). Then, 150 μl of FD4 (80 mg/mL) was gavaged orally to each rat, and serum was collected 1 h later. The serum concentration of FD4 was measured using a FLUOstar Optima plate reader (BMG Labtech, France) with an excitation wavelength at 490 nm and an emission wavelength of 525 nm as previously reported [28].

#### (3) Evaluation of colonic damage

The animals were sacrificed by cervical dislocation on day 5 after the blood sample collection. The severity of colonic damage was assessed by an independent observer who was blinded from the treatments. The entire colon was excised and thoroughly observed for gross examination. A distal 10 cm portion of the colon was cut open longitudinally, washed with physiological saline to remove the faecal residues, and imaged the colon morphology and weighed to calculate the weight to length ratio. The colonic damage scores were assigned according to a macroscopic scoring system [29]. The disease activity index was calculated based on stool consistency, rectal bleeding, and weight loss percentage [30]. The colon samples were frozen immediately in liquid nitrogen and stored at -80 °C for further biochemical, and western blot studies.

#### (4) Biochemical Assays

The expression levels of proinflammatory cytokines and mediators, such as TNF-α, secretory IgA and myeloperoxidase in the rat colon were determined using ELISA kits according to the manufacturer’s protocol.

#### (5) Histology

The distal colons were isolated on day 5 after the induction of colon inflammation and fixed in 10% neutral buffered formalin for 24 h and embedded in paraffin. Colon sample sections were prepared and stained with hematoxylin and eosin (H&E) and then assessed under light microscopy. The histological damage score was calculated according to Hunter et al. [31] on a 12-point scale: loss of architecture, 0–3; inflammatory infiltrate, 0–3; goblet cell depletion, 0 or 1; ulceration, 0 or 1; oedema, 0 or 1; muscle thickening, 0–2; and presence of crypt abscesses, 0 or 1. Colon microscopic scoring was performed by an experienced pathologist who was blinded to the study design.

#### (6) Western blot Analysis

The colon tissue samples were homogenized with lysis buffer and incubated for 15 min on ice. Further, the samples were centrifuged for 5 min at 12,000 r.p.m. at 4˚C and the extracted protein were quantified using BCA protein assay kit. Equal amounts of protein were subjected to Western blot analysis as previously described [32]. Protein lysates were separated by 10% sodium dodecyl sulfate polyacrylamide gel electrophoresis (SDS-PAGE) and transferred to polyvinylidene difluoride (PVDF) membranes. Membranes were blotted for specific antibodies, such as occludin, ZO-1 & claudin-2. Finally, the antibody bound protein bands were visualized using an enhanced chemiluminescence detection kit in ChemiDoc image scanner. Quantification was performed by densitometric analysis of specific bands on the immunoblots using ImageJ software.

## Statistical analysis

All the data are shown as the mean ± standard deviation (SD). Statistical analysis was performed using one-way analysis of variance (ANOVA), followed by post hoc Bonferroni/Dunnett’s test. *P*-value < 0.05 was considered as statistically significant.

## Results

### In vitro intestinal barrier dysfunction study

#### Cell viability at FREG administration

**Fig. 1:**
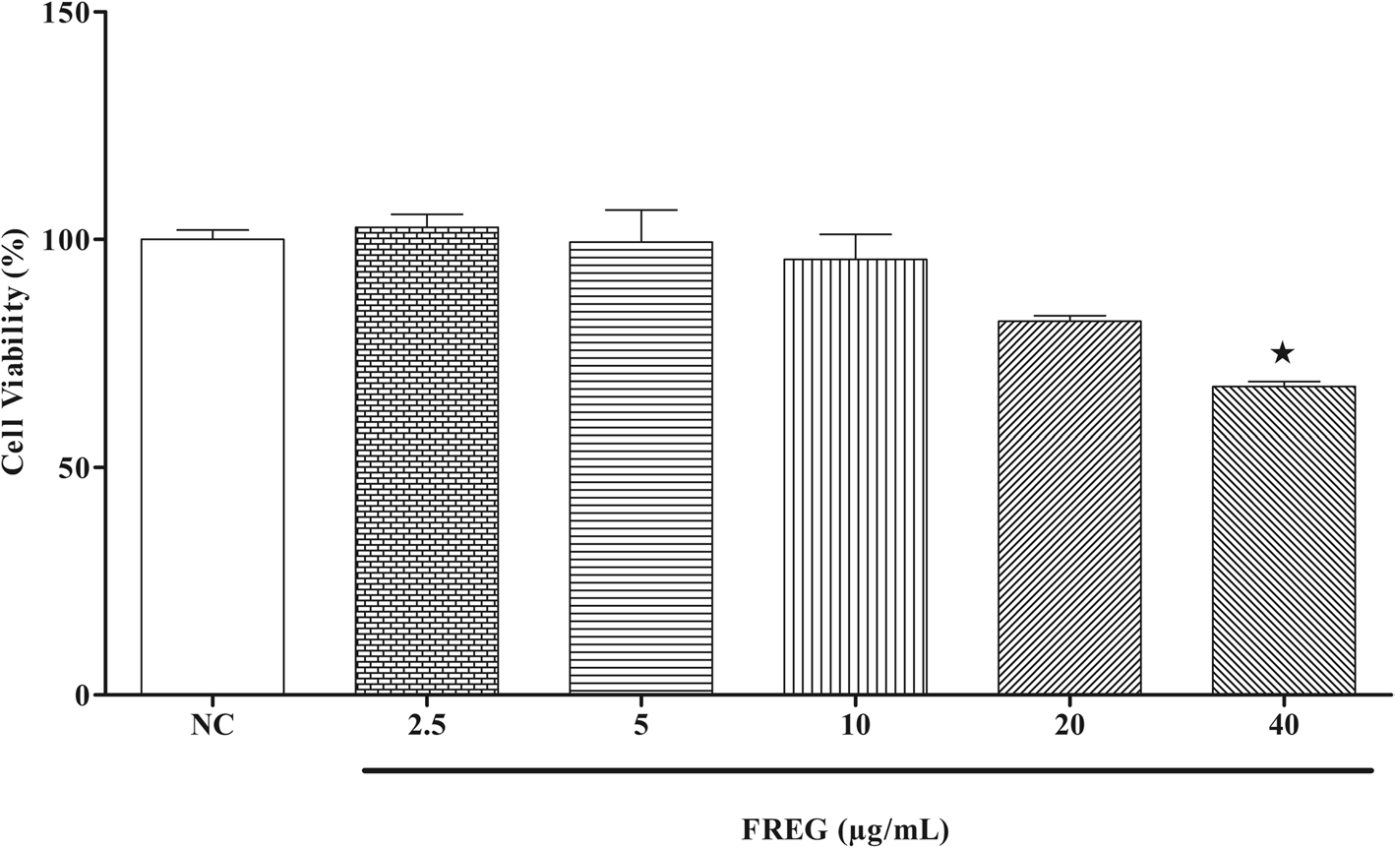
Cytotoxic effect of FREG on Caco-2 cells. Caco-2 cells were treated with different concentrations of FREG for 48 h at 37 °C. Caco-2 cells viability was measured by MTT assay. Values are presented as mean ± standard deviation of the mean of three replicates. An asterisk indicates a significant (^**★**^*p* < 0.05) difference from cell control

The cytotoxicity of FREG in human intestinal epithelial cells was examined in vitro to determine the effective concentration with no toxicity for subsequent experiments. The results showed that the exposure of FREG at 2.5–20 μg/mL for 48 h had no significant effect on the viability of human intestinal epithelial cells (Fig. 1).

### Effect of FREG in preventing and restoring the intestinal barrier dysfunction

**Fig. 2:**
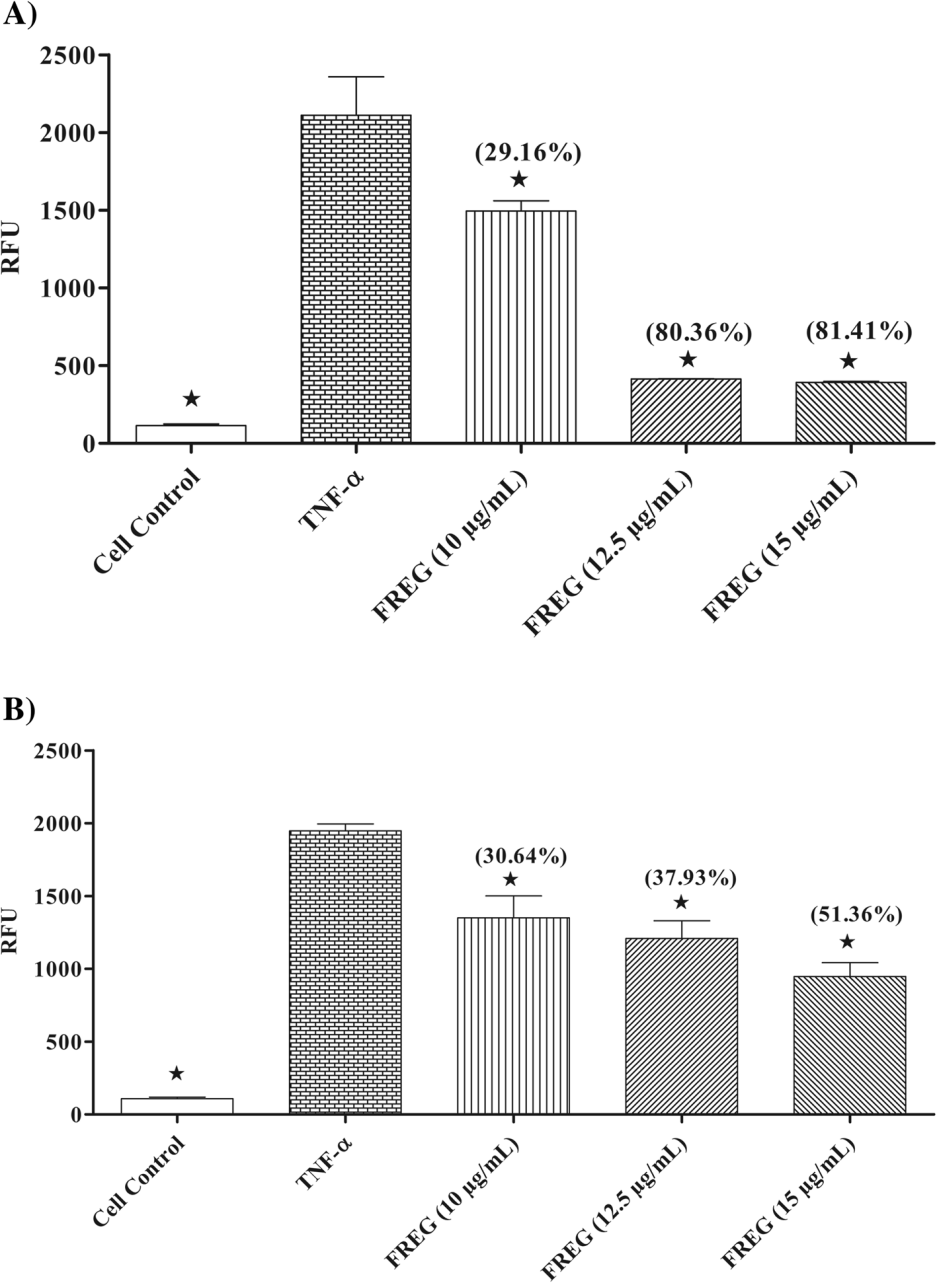
**A** Preventive effect of FREG on TNF-α induced intestinal barrier dysfunction. Caco-2 cell monolayers were treated with different concentration of FREG, and barrier dysfunction was induced with TNF-α (20 ng/mL) and then assessed for paracellular permeability to a tracer molecule FD-4. ^**★**^*P* < 0.05, TNF-α control Vs treated groups. Values in the parentheses represent the percentage decrease compared to TNF-α control. RFU: Relative Fluorescence Units. **B** Curative effect of FREG on TNF-α induced intestinal barrier dysfunction. Caco-2 cell monolayers were treated with TNF-α (20 ng/mL) to induce barrier dysfunction. After 24 h incubation, cells were washed with prewarmed PBS and incubated with different concentration of FREG for 24 h. Paracellular permeability was assessed by using a tracer molecule FD-4. ^**★**^*P* < 0.05, TNF-α control Vs treated groups. Values in the parentheses represent the percentage decrease compared to TNF-α control. RFU: Relative Fluorescence Units

FREG attenuated the disruption of intestinal epithelial barrier function induced by TNF-α in Caco-2 cell monolayers. Caco-2 cell monolayers were treated with FREG (10, 12.5 and 15 µg/mL) and TNF-α (20 ng/mL) and then was assessed for paracellular permeability to a tracer molecule FD-4. TNF-α increased the permeability of a tracer molecule FD-4 in Caco-2 cell monolayers and this increased paracellular permeability of FD-4 was significantly suppressed by FREG in both cotreatment as well as curative methods. The percentage decrease of paracellular flux to FD-4 were presented in the Fig. 2a and b.

### Effects of FREG on intestinal barrier dysfunction in vivo

**Fig. 3:**
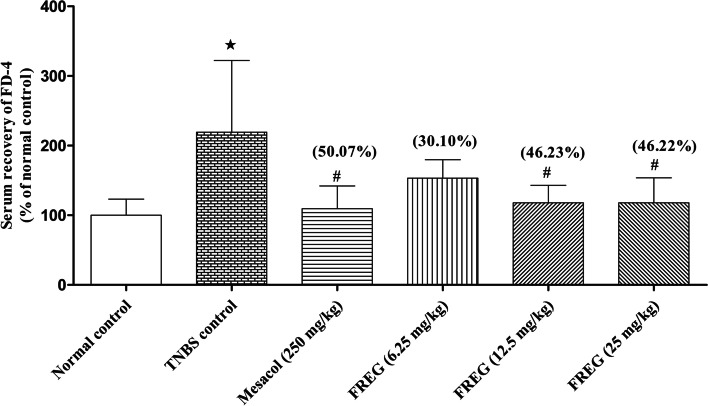
Effect of FREG on TNBS induced intestinal barrier dysfunction in rats. After 5 days of FREG administration to rats, the intestinal permeability in each group was examined through serum recovery of FD-4. Data were expressed as the mean ± SD; *n* = 6. ^★^*P* < 0.05 significantly different from normal control group. ^**#**^*P* < 0.05 significantly different from TNBS control groups. Values in the parenthesis represent percentage decrease of FD-4 leakage compared to TNBS control group

TNBS significantly disrupted the intestinal epithelial barrier function as evident from significantly increased recovery of serum FD-4 in TNBS-treated rats compared with vehicle treated rats. Both the positive control Mesacol (250 mg/kg) and FREG (12.5 & 25 mg/kg) significantly decreased the serum recovery of FD-4 compared to TNBS-treated rats (Fig. 3) suggest that intestinal epithelial barrier dysfunction is recovered by FREG administration.

### Effect of FREG on TNBS induced alteration in colon weight to length ratio, Macroscopic score and Disease activity index (DAI) in in vivo

**Fig. 4:**
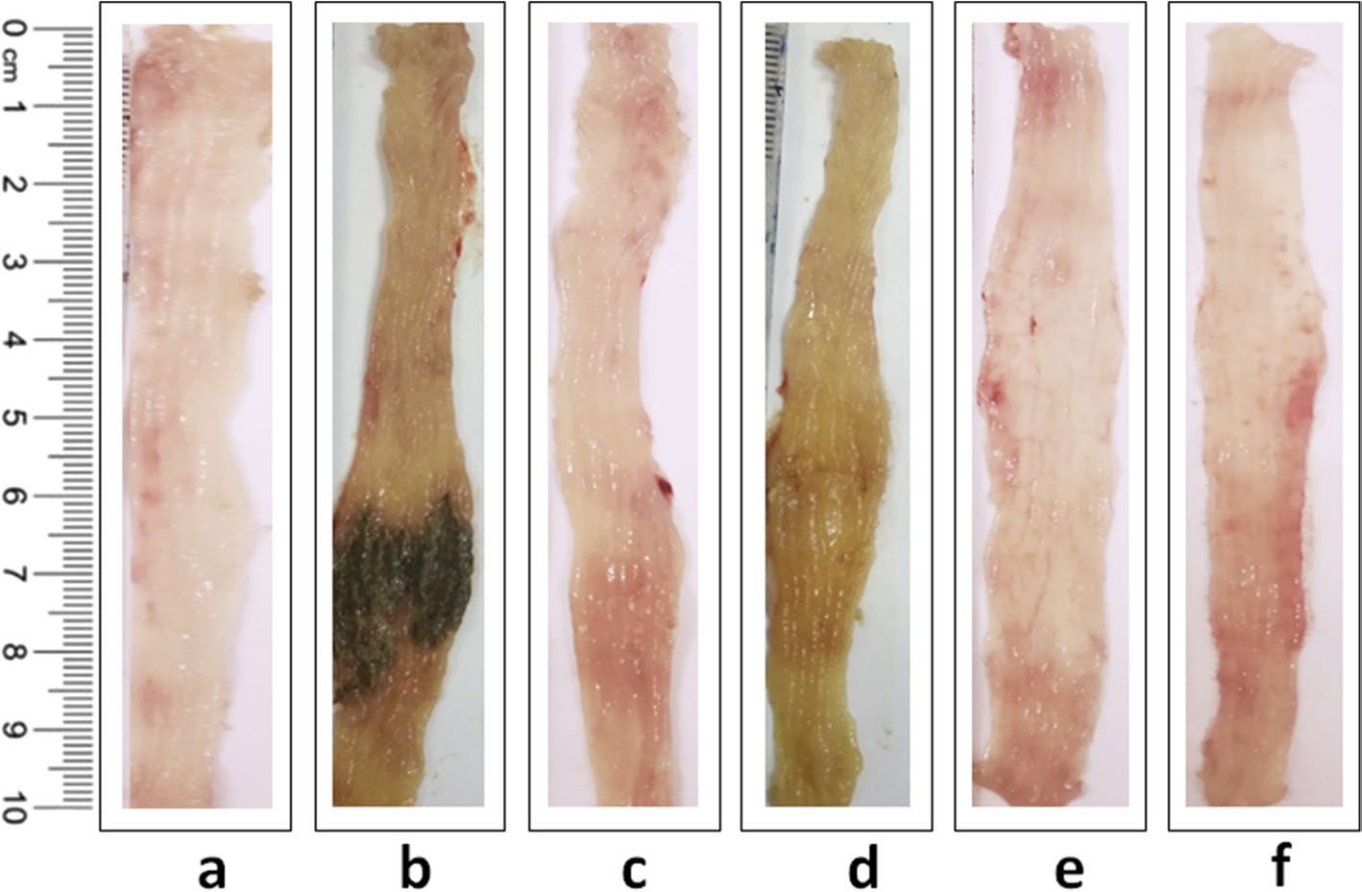
Effect of FREG on TNBS induced alterations in colon morphology. (**a**) Normal control, (**b**) TNBS control, (**c**) Mesacol (250 mg/kg), (**d**) FREG (6.25 mg/kg), (**e**) FREG (12.5 mg/kg), and (f) FREG (25 mg/kg)

In comparison to the normal control rats, colon weight to length ratio, DAI and macroscopic scores were increased significantly after intrarectal administration of TNBS to rats. Administration of Mesacol (250 mg/kg) significantly decreased colon weight to length ratio, macroscopic score and DAI scores in comparison to that of the TNBS control rats. Similarly, colon weight to length ratio, macroscopic and DAI scores were decreased significantly after treatment with FREG at all the dose levels except 6.25 mg/kg for DAI scores (Table 1). Morphological representation of colons from different treatment groups were presented in Fig. 4.

**Table 1:** Effect of FREG on TNBS induced alterations in colon weight to length ratio, macroscopic scores & DAI scores in rats. Colon weight to length ratio, macroscopic and DAI scores were decreased significantly after treatment with FREG. Data were expressed as the mean ± SD; *n* = 6. ^★^*P* < 0.05 significantly different from normal control group. ^#^*P* < 0.05 significantly different from TNBS control group

Treatment Groups	Colon weight to length ratio (mg/cm)	Macroscopic scores	DAI scores
Normal control	101.48 ± 5.03	0.17 ± 0.41	0.00 ± 0.00
TNBS dose 25 mg	180.72 ± 22.49^★^	4.00 ± 1.26^★^	1.33 ± 0.0^★^
Mesacol 250 mg/kg	134.12 ± 8.47^#^	0.50 ± 0.55^#^	0.11 ± 0.27^#^
FREG 6.25 mg/kg	123.53 ± 9.73^#^	1.00 ± 0.89^#^	0.39 ± 0.61
FREG 12.5 mg/kg	133.07 ± 13.94^#^	0.50 ± 0.55^#^	0.22 ± 0.54^#^
FREG 25 mg/kg	142.17 ± 22.10^#^	0.50 ± 0.55^#^	0.22 ± 0.35^#^

### Effect of FREG on TNBS induced intestinal inflammation and modulation of mucosal immunity in vivo

**Fig. 5:**
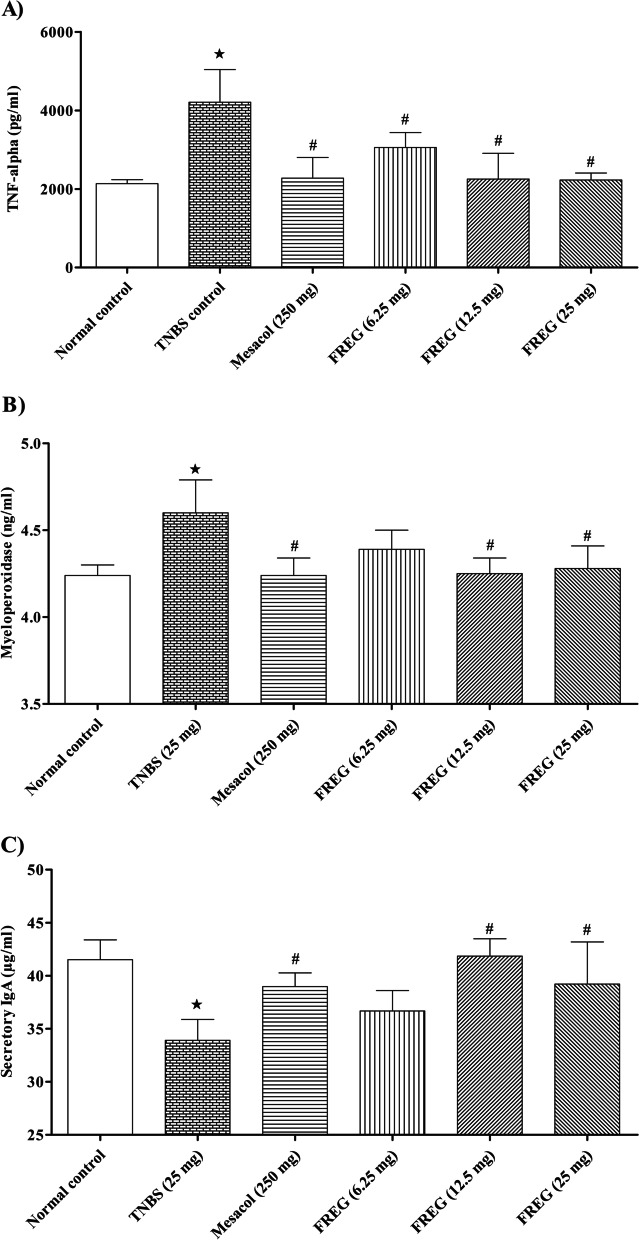
Effect of FREG on inflammatory and mucosal immunity markers in TNBS-treated rats colon. (**A**) Effect of FREG on the expression of TNF-α, (**B**) Effect of FREG on MPO activity and (**C**) Effect of FREG on mucosal immunity (SIgA) in TNBS-treated rat colon. Data were expressed as the mean ± SD; *n* = 6. ^**★**^*P* < 0.05 significantly different from normal control group. ^#^*P* < 0.05 significantly different from TNBS control groups

The present study indicated that levels of proinflammatory cytokine such as TNF-α and oxidative stress marker MPO activity in the resected colon from TNBS-treated rats were significantly higher than in the normal control rats. Administration of Mesacol and FREG (12.5 & 25 mg/kg) significantly decreased TNF-α and MPO activity compared with TNBS-treated rats (Fig. 5A and B). SIgA plays a crucial role in the immune function of mucosal epithelial membranes. Our study results showed that the level of SIgA in the resected colon from TNBS-treated rats were significantly lower compared with vehicle control rats. Administration of Mesacol and FREG (12.5 & 25 mg/kg) significantly increased the SIgA levels when compared to TNBS-treated rats (Fig. 5C).

### Effect of FREG on TNBS induced histological alterations in colon tissue of rats

Figure 6a depicts the normal structure of colon tissue. Histological examination of the colon tissues revealed a significant increase of inflammatory cell infiltrate, loss of colonic architecture, oedema, muscle thickening, goblet cell depletion and transmural ulceration in TNBS control rats (Fig. 6b) in comparison to the normal control rats. However, administration of Mesacol (250 mg/kg) significantly attenuated TNBS induced histological alterations (Fig. 6c) when compared with TNBS control rats. In comparison to the TNBS control rats, the administration of FREG (6.25, 12.5 & 25 mg/kg) also significantly decreased the inflammatory cell infiltrate, prevented the loss of colonic architecture, protected the goblet cell depletion, and reduced the transmural ulceration and oedema in the colon tissues (Fig. 6d, e, and f). The overall histological damage score was significantly higher in TNBS control rats in compared to the normal control rats. Treatment with Mesacol and FREG at all the dose levels were significantly decreased the histological damage score when compared to TNBS control group (Fig. 6g).

**﻿Fig. 6:**
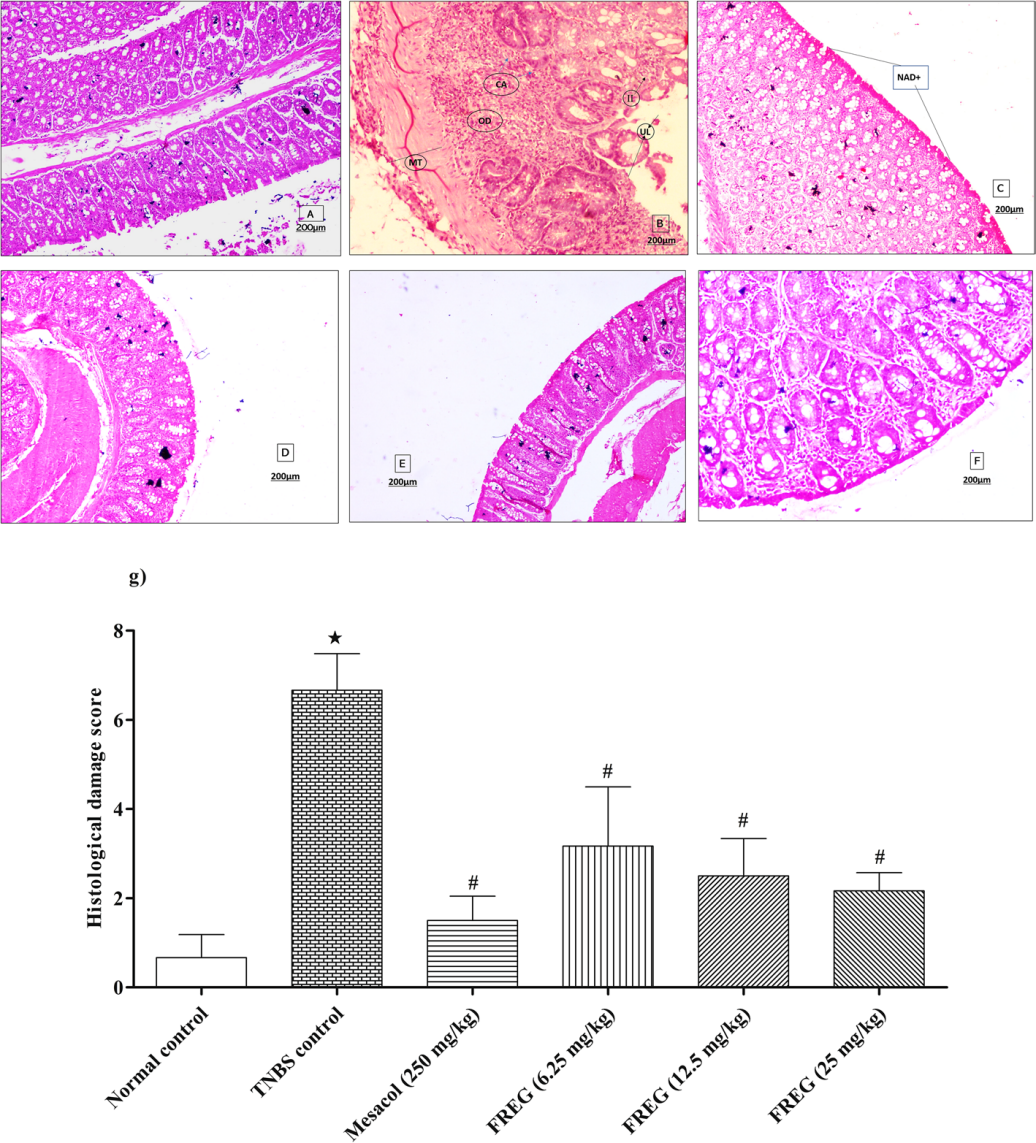
Effect of FREG on TNBS induced alterations in colon histopathology. Photomicrograph of sections of rat colon stained with H&E stain (**A**) Normal control: showing normal morphology (NAD+) (X100), (**B**) TNBS control: UL-Ulceration, II-Inflammatory infiltrate, CA-Crypt Abscess, OD-oedema, MT-Submucosal Thickening (X50), (**C**) Mesacol (250 mg/kg): showing normal morphology observed (NAD+) (X50), (**D**) FREG (6.25 mg/kg: showing normal morphology observed (NAD+) (X100), (**E**) FREG (12.5 mg/kg): showing normal morphology observed (NAD+) (X100), and (**F**) FREG (25 mg/kg): showing normal morphology observed (NAD+) (X100). (**G**) The quantitative representation of histological score. Data were expressed as the mean ± SD; *n*=6. ★*P* < 0.05 significantly different from normal control group. #*P* < 0.05 significantly different from TNBS control groups. Scale bar = 200 µm

### Effect of FREG on TNBS induced alterations of tight junction proteins in colon tissue of rats

**Fig. 7:**
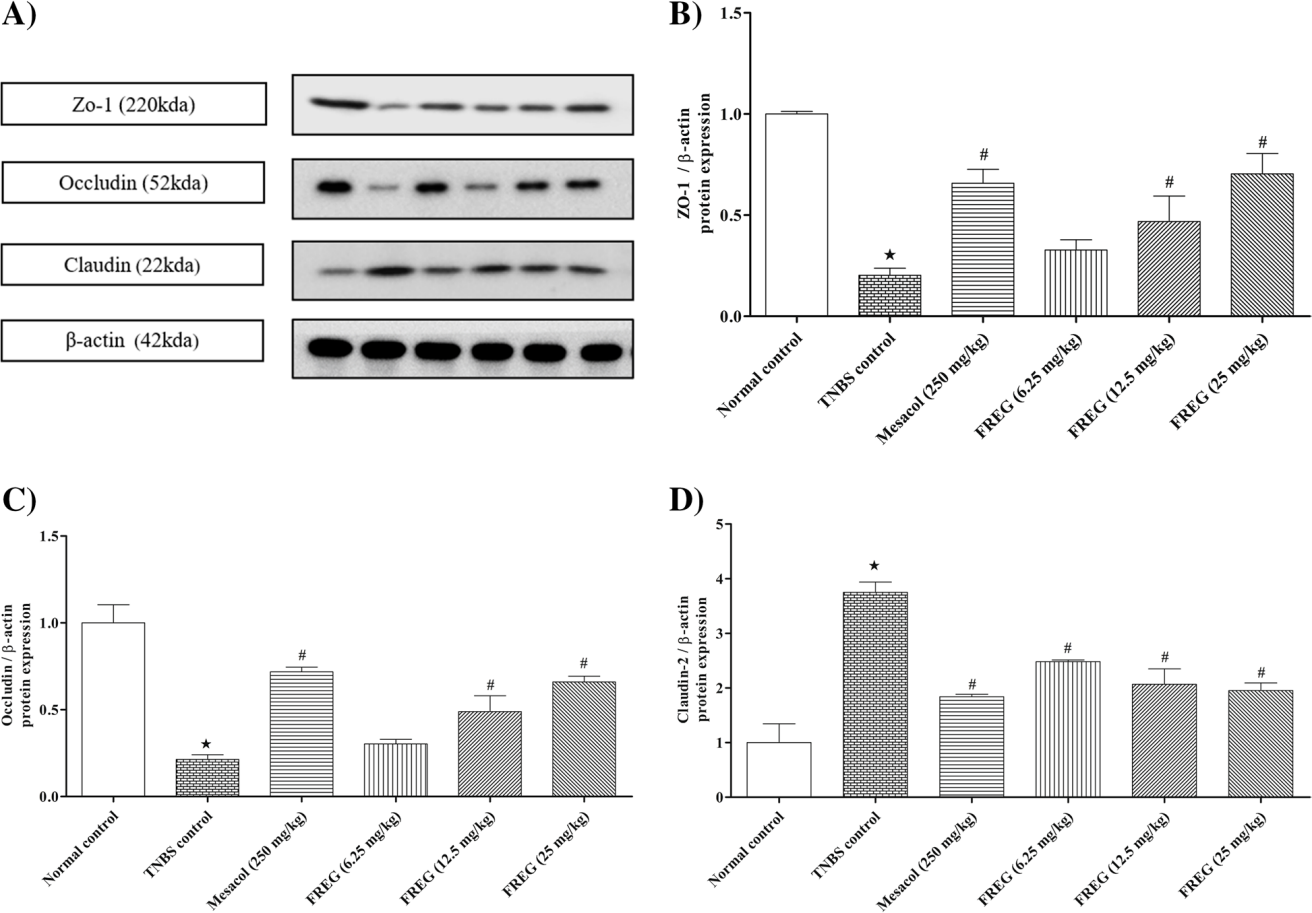
Effect FREG on tight junction proteins (ZO-1, Occludin & Claudin-2) in rat colon. FREG administration increased the expression of tight junction proteins such as ZO-1 & occludin and decreased the pore-forming protein claudin-2. **A**) Western blot of one representative experiment. **B**, **C** and **D**) Densitometric data of three independent experiments. Data were expressed as the mean ± SD. ^**★**^*P* < 0.05 significantly different from normal control group. ^#^*P* < 0.05 significantly different from TNBS control groups

Intrarectal administration of TNBS resulted in significant downregulation of colonic occludin and ZO-1 protein expressions in TNBS control rats compared to the normal control rats. When compared to TNBS control rats, treatment with Mesacol (250 mg/kg) substantially prevented the downregulation of colonic occludin and ZO-1 protein expression. The FREG (6.25, 12.5 & 25 mg/kg) treatment showed substantially increased colonic occludin and ZO-1 protein expression in comparison to the TNBS control rats (Fig. 7). Concomitantly, the expression of a pore-forming protein claudin-2 was increased in TNBS-treated rats compared to the normal control rats. Mesacol and FREG treatments substantially reduced the claudin-2 protein expression compared to the TNBS control rats (Fig. 7).

## Discussion

The intestinal epithelium is a major component of gut barrier, supporting two important functions facilitating the nutrients absorption, and controlling the passage of pathogens and/or toxins from the gut lumen into the blood circulation. The intestinal mucosal barrier is mainly formed and maintained by a single layer of proliferating and differentiating intestinal epithelial cells (IECs). IECs constitute an efficient barrier, maintained by the network of complex proteins that mechanically connects the neighbouring cells and regulates the passage of luminal contents such as pathogens and/or toxins that pass through the epithelial layer by either between the cells (paracellular flux) or through the cells (transcellular flux) into the mucosa. Tight junctions and its proteins are major component in this network which controls the intestinal permeability. Alterations in the tight junction protein formation and distribution and/or destabilization of the tight junction complexes leads to intestinal epithelial barrier dysfunction [33]. Therefore, the integrity of the epithelial monolayer is critical for the gut, and epithelial barrier dysfunction is a hallmark of various intestinal disorders such as ulcerative colitis, Crohn’s disease, irritable bowel syndrome, celiac disease and infectious diarrhoea & etc. leading to metabolic, autoimmune, and psychological disorders [34].

The intestinal epithelial barrier dysfunction is characterized by an increase in gut permeability and defects in the intestinal epithelial barrier repair. Reducing the barrier dysfunction, or enhancing the mucosal repair processes, are increasingly recognized as being of major therapeutic interest [35, 36]. The aetiology of the intestinal barrier dysfunction has been attributed to several factors, among which mucosal inflammation induced by proinflammatory cytokines play critical role. It is well reported that proinflammatory cytokines such as TNF-α disrupts the intestinal epithelial barrier functions both in vitro and in vivo, [37, 38] causing intestinal disorders. Thus, targeting the restoration of intestinal barrier function is a key therapeutic strategy in acute or chronic enteropathies.

In recent times, research has been focused on understanding the role of phytoconstituents in particularly flavonoids in modulating the inflammatory response and protecting the gut barrier integrity [39]. The investigational test substance FREG is a flavonoid rich G*lycyrrhiza glabra* extract that has been found to possess Anti-inflammatory, antioxidant, antiulcer, and anti-*Helicobacter pylori* activities [20–22]. FREG also has significant inhibitory activity against the release of proinflammatory mediators [23]. Thus, the present study was performed to investigate the effects of FREG on protecting the intestinal barrier function in TNF-α﻿ induced epithelial barrier dysfunction in human Caco-2 cells and in TNBS induced colitis rats.

In in vitro study, FREG attenuated the intestinal barrier dysfunction induced by TNF-α﻿﻿ in Caco-2 cell monolayers as evident from significant decrease in paracellular permeability to FD-4. These observations indicate that FREG has the potential to protect the gut barrier function against proinflammatory cytokine-mediated barrier dysfunction. The ability of FREG to limit the TNF-α﻿ induced increase in epithelial permeability is probably attributed to the antioxidant and anti-inflammatory activity of FREG [23]. To further confirm these results, we examined the effects of FREG on the epithelium barrier dysfunction in vivo and found that FREG reduced the permeability of the intestine and protected the tight junctions and its proteins in the colon of TNBS induced rats.

In the in vivo study, intestinal barrier disruption was induced by intracolonic administration of TNBS in rats. TNBS can modify the colon tissue proteins by forming a covalently reactive compound and act as a hapten that produces immunological responses to the modified colon tissue proteins which further leads to the damage of intestinal epithelium barrier [40]. In the present investigation, FREG ameliorated the TNBS induced epithelial barrier dysfunction in colitis rats. TNBS induced intestinal barrier dysfunction is found to result in increased intestinal permeability characterized by enhanced serum recovery of FD-4 [41]. Our results revealed that the intestinal permeability to FD-4 was significantly lower in the FREG administered group (46%) compared to TNBS control group, suggesting that FREG prevents the increase of intestinal permeability thereby protecting the gut barrier integrity. The potential protective effect of FREG could be attributed to its flavonoids, the major constituents, as many naturally occurring bioactive flavonoids have been reported to exert beneficial effect over the intestinal barrier function [42, 43].

We also examined the severity and extent of the inflammatory response by measuring weight to length ratio of the inflamed colon, DAI, macroscopic and microscopic analysis. In this investigation, intrarectal administration of TNBS caused elevated inflammatory response as evident from increase in colon weight to length ratio which confirms the intensification of intestinal infiltrations and subsequent intestinal oedema [44]. The disease activity index and macroscopically visible damage were significantly higher in the TNBS-treated rats. All these changes were reversed significantly with FREG administration and the effects were comparable to a positive drug Mesacol. Microscopic examination of colon from rats in the TNBS group revealed epithelial cell necrosis, oedema, ulceration, and neutrophil infiltration. This is due to the fact that the released proinflammatory cytokines such as TNF-α caused enhanced synthesis of PGE2 and exacerbated tissue damage [45]. FREG protected against these alterations and reduced the histopathological scores revealing attenuated inflammatory cell infiltration and preservation of colon architecture while oedema was still detected. These results are in accordance with our earlier findings [21]. In addition to the in vitro study on FREG, the published evidence by Kwon et al. [46] on glabridin corroborates the protective effects on the gut via anti-inflammatory mechanisms.

The release of inflammatory cytokines and infiltration of neutrophils play an important role in colon inflammation [45]. In this study, TNBS causes elevated colonic levels of TNF-α and MPO. Administration of FREG significantly decreased the production of TNF-α in a dose dependent manner. Probably the decreased release of TNF-α caused reduction in cell infiltration and mucosal ulceration in FREG treated groups. The significant inhibitory effect of FREG on the release of inflammatory mediators was also reported in our earlier studies [21, 23]. Progressive inflammation activates infiltration of inflammatory cells that triggers further pathological responses [47]. Leukocyte invasion into the colonic tissues was confirmed by increased activity of MPO, a biochemical index for neutrophil influx [48]. The current study showed that TNBS treatment increased the levels of colonic MPO, indicating neutrophil infiltration. FREG significantly ameliorated colonic MPO activity plausibly by decreasing inflammatory cells invasion that was evident in the histological findings similar to that observed by Kwon et al. [46]. The study also demonstrated FREG exerts a protective effect on mucosal immunity by markedly increasing the colonic SIgA levels. SIgA plays a key role in the first line of immune defence by protecting the mucosal surfaces against environmental pathogens and commensal bacteria resulting in a beneficial downregulation of inflammation [49].

The tight junction complexes play a major role for the maintenance of epithelial barrier integrity. It is a multiprotein complex which comprises of transmembrane proteins such as occludin and claudins, peripheral membrane proteins such as zonula occludens and regulatory molecules [50]. Recent research has shown that the integrity of tight junction is dependent on the number, composition, and mixing ratio of claudins, while occludin plays a significant role in the assembly and maintenance of the tight junctions [51]. Structural modification to these colonic tight junction proteins due to an elevated oxidative stress or inflammatory response result in upregulation of claudin-2 and downregulation of occludin, and ZO-1 expressions. In turn, this leads to an increase in intestinal permeability and intestinal barrier dysfunction [26]. The present investigation revealed that intrarectal instillation of TNBS, decreased the expression of occludin, and ZO-1 proteins while increased the expression of claudin-2, a pore-forming protein responsible for increased paracellular permeability. These observations are in accordance with the earlier reported studies [26, 52]. All these changes were reversed by treatment with FREG, demonstrating its protective role against intestinal barrier dysfunction.

## Conclusion

The present study revealed that FREG ameliorated TNF-α and TNBS induced barrier dysfunction in vitro and in vivo respectively. FREG demonstrated beneficial effects by reducing MPO activity, inhibiting pro/inflammatory cytokine release and modulating the expression of claudin-2, occludin and ZO-1 proteins in the tight junctions thereby protecting the intestinal barrier integrity. In addition, FREG increased the colonic SIgA levels indicating its beneficial effects on mucosal immunity. These studies demonstrated the potential of flavonoid rich extract of licorice (Gutgard) in various gut related health benefits.

## Supplementary Information

**Additional file 1:** **Supplementary Figure S1.** The unprocessed immune blot data of individual tight junction proteins from experimental rats colon tissue sample.

## Data Availability

All data generated or analyzed during this study are included in this published article [and its supplementary information files].

## Abbreviations

FREG: Flavonoid rich extract of *Glycyrrhiza glabra*
TNF-α: Tumor necrosis factor-alpha
TNBS: 2,4,6-Trinitrobenzenesulfonic acid
FD4: FITC-conjugated 4-kD dextran
MPO: Myeloperoxidase
SIgA: Secretory immunoglobulin A
IECs: Intestinal epithelial cells
ZO: Zonula occludens
INF-γ: Interferon-gamma
ILs: Interleukins
ROS: Reactive oxygen species
COX: Cyclooxygenase
LOX: Lipoxygenase
IBS: Irritable bowel syndrome
LPS: Lipopolysaccharide
DMEM: Dulbecco's Modified Eagle Medium
FBS: Fetal bovine serum
MTT: 3-(4,5-Dimethylthiazol-2-yl)-2,5-diphenyltetrazolium bromide
PBS: Phosphate buffered saline
CO_2_: Carbon dioxide
CMC: Carboxymethyl cellulose
NaCl: Sodium chloride
IAEC: Institutional Animal Ethics Committee
DAI: Disease activity index
ELISA: Enzyme-linked immunosorbent assay
PGE_2_: Prostaglandin E_2_
