# Supplementary material for: A flavonoid rich standardized extract of Glycyrrhiza glabra protects intestinal epithelial barrier function and regulates the tight-junction proteins expression
Source: BMC Complement Med Ther. 2022 Feb 7;22:38. doi: 10.1186/s12906-021-03500-1 (PMC8822647; doi:10.1186/s12906-021-03500-1)
Supplement: Supplementary file 1 — Additional file 1: Supplementary Figure S1. The unprocessed immune blot data of individual tight junction proteins from experimental rats colon tissue sample. [file 12906_2021_3500_MOESM1_ESM.doc]

**Supplementary Information**

**A flavonoid rich standardized extract of Glycyrrhiza glabra protects intestinal epithelial barrier function and regulates the tight-junction proteins expression**

**Editorial requests:**

If original images of full-length blots cannot be provided, please include images of all blots as they are, with membrane edges visible, and for all replicates performed in the Supplementary Information file and include an explanation for the absence of images of adequate length where appropriate in the manuscript.

**Authors Explanation:**

We agreed that the given western blot was cropped from the same full-length blots. The reason is to removes irrelevant parts of the captured image and presents only the proteins of interest. And also, the membrane itself may be spliced after protein transfer and stained by Ponceau Red and then probed with interest of antibodies to reduce the volume of reagents/antibody needed for the experiment. However, a gel itself is never spliced. we only cropped the captured image to present the area of interest.

As per the requests from the editor during the revision, here we have presented the Original images of all blots as it is after hybridization with antibodies performed for our study.

**Supplementary Figure: 1**


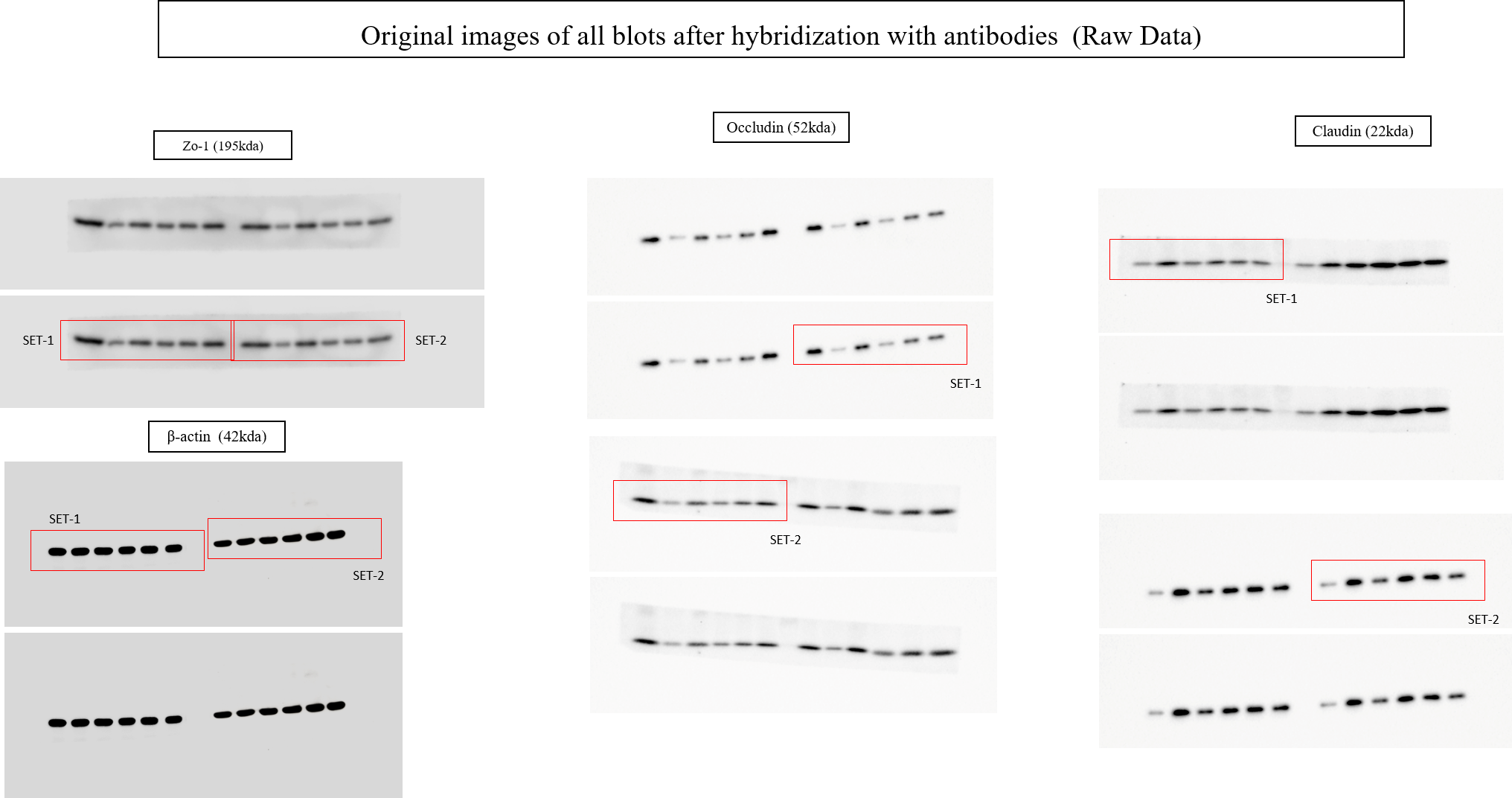
The unprocessed immune blot data of individual tight junction proteins from experimental rats colon tissue sample.

**Legend:**

Figure 1. Immunoblots represents the tight-junction proteins such as ZO-1, Occludin and Claudin-2 expression were assessed to evaluate the protective effect of a flavonoid rich extract of Glycyrrhiza glabra (FREG) on epithelial barrier function upon intestinal inflammation. The western blot results clearly reveal that FREG has greatly alter the paracellular permeability, thereby reducing the gut barrier dysfunction. The TNBS administered (Group 2) rats showed significant downregulation of colonic occludin and ZO-1 protein expressions and significant upregulation of claudin-2 expression when compared to the control (Group 1) rats. Whereas the FREG treated (Group 4, 5 and 6) rats showed significant upregulation of colonic occludin and ZO-1 protein expression and significant downregulation of claudin-2 protein expression when compared with TNBS administered (Group 2) rats. A positive drug mesacol treated (Group-3) rats also showed similar effects.
